# Comparing the diagnostic accuracy of pre-operative genetic testing for thyroid cancer on fine needle aspiration cytology specimens: a systematic review and meta-analysis of diagnostic accuracy

**DOI:** 10.1007/s12672-025-03676-9

**Published:** 2025-10-02

**Authors:** Xin Liao, Klaas Van Den Heede, Bruno Lapauw, Wouter Huvenne, Dirk Ysebaert, Vanessa Meert, Ying Fang, Jing Li, Sam Van Slycke, Nele Brusselaers

**Affiliations:** 1 Global Health Institute, Faculty of Medicine and Health Sciences, University of Antwerp, Antwerp, Belgium; 2 Department of General and Endocrine Surgery, AZORG Hospital Aalst, Moorselbaan 164, Aalst, 9300 Belgium; 3 Department of Internal Medicine and Paediatrics, Department of Endocrinology, Ghent University Hospital, Ghent University, Ghent, Belgium; 4 Department of Head and Skin, Ghent University Hospital, Ghent University, Ghent, Belgium; 5 Department of Hepatobiliary, Transplantation and Endocrine Surgery, Antwerp University Hospital, Antwerp, Belgium; 6 Department of Pathology, AZORG Hospital Aalst, Aalst, Belgium; 7 Department of Operating Theatre, Yueyang Central Hospital, Yueyang, Hunan China; 8 Department of Cardiac Surgery, Yueyang Central Hospital, Yueyang, Hunan China; 9 Department of Public Health and Primary Care, Ghent University, Ghent, Belgium; 10 Department of Women’s and Children’s Health, Karolinska Institutet, Stockholm, Sweden

**Keywords:** Thyroid cancer, Indeterminate thyroid nodule, Diagnostic accuracy, Meta-analysis

## Abstract

**Background:**

Molecular genetic tests are increasingly used to determine the need for surgery in thyroid nodules with indeterminate fine-needle aspiration (FNA) cytopathology. However, the accuracy of these tests remains uncertain. This systematic review and meta-analysis analyze the diagnostic performance of molecular testing in pre-operative FNA biopsies from indeterminate thyroid nodules (ITN).

**Methods:**

We searched PubMed, Embase, Web of Science, and the Cochrane Library for relevant studies. The preoperative FNA cytopathology from patients over 14 years old, and the postoperative histopathology results were extracted. It was also essential to include true-positive (TP), false-positive (FP), true-negative (TN), and false-negative (FN) counts, along with genetic testing results. Sensitivity, specificity, diagnostic odds ratio (DOR), positive likelihood ratio (PLR), negative likelihood ratio (NLR), and area under the curve (AUC) were calculated for each molecular panel using a random-effects bivariate model.

**Results:**

Overall, 68 studies (2018–2024) were eligible discussing 7 different panels, from 16 countries. Multigene Point-of-care Test (MPTX v1) demonstrated the strongest ability to rule out malignancies (NLR 0.12; *n* = 4) and exhibited the highest diagnostic value (DOR 18; *n* = 4). ThyroSeq v2 Next-Generation Sequencing Test for Thyroid Cancer (ThyroSeq v2) followed with a DOR of 10 (*n* = 9). The PCR groups(*n* = 20) could not be merged due to significant methodological heterogeneity.

**Conclusion:**

This meta-analysis highlights the role of molecular testing in improving the diagnostic accuracy of indeterminate thyroid nodules, potentially reducing unnecessary surgeries. However, further standardization and validation are needed due to study heterogeneity.

**Supplementary Information:**

The online version contains supplementary material available at 10.1007/s12672-025-03676-9.

## Introduction

Thyroid cancer is the most prevalent endocrine malignancy worldwide, with an estimated 586,000 cases diagnosed in 2020 [1]. Although recent advancements in high-resolution ultrasonography and Fine-Needle Aspiration biopsy (FNA) have led to improved malignant thyroid nodule detection, many nodules remain indeterminate. Bethesda categories III, IV, and V represent indeterminate thyroid nodules that cannot be definitively classified as benign or malignant based on cytology alone [2, 3]. These include follicular lesions of undetermined significance (FLUS; Bethesda III), atypia of undetermined significance (AUS), follicular neoplasm or suspicious follicular neoplasm (SFN; Bethesda IV), and suspicious malignant tumors (SM; Bethesda V). Consequently, a substantial proportion of patients with indeterminate nodules undergo potentially unnecessary surgery, as only 20% to 30% are ultimately confirmed as malignant on final histopathological examination [4]. 

To address the limitations of cytology-based diagnoses, molecular testing techniques have emerged, focusing on RNA-based gene expression analysis and the detection of genetic mutations such as copy number alterations, point mutations, insertions, and deletions [5–7]. These advances permit a more exact evaluation of the malignant potential of indeterminate thyroid nodules, thus reducing the frequency of unnecessary surgery and corresponding morbidity and additional medical costs.

Initially driven by single-gene analysis, the study of indeterminate thyroid nodules now includes comprehensive next-generation sequencing approaches that analyze both DNA and RNA [8, 9]. Widely used molecular assays for thyroid nodules include Afirma Gene Sequencing Classifier (Afirma GSC)/ Afirma Xpression Atlas Gene Expression Profile (Afirma Xpression Atlas), Multiplex Polymerase Chain Reaction Test (MPTX v1), and Next-Generation Sequencing Test for Thyroid Cancer (ThyroSeq v2), which have demonstrated enhanced diagnostic accuracy when applied to routinely processed FNA samples [10]. These assays use various molecular markers such as *BRAF*^*V600E*^ mutations, *RET* fusions, and *TERT* promoter mutations to predict the likelihood of malignancy and inform clinical decision-making [11–13]. 

The ThyroSeq 2 genetic test, for instance, employs next generation sequencing to analyze 112 genes for genetic alterations and employs genomic classifiers to distinguish between benign and malignant lesions [14]. Such genetic testing platforms are increasingly integrated into clinical practice to improve the diagnostic accuracy of indeterminate thyroid nodules [15]. 

Several authors have done diagnostic meta-analyses of commercially available genetic testing panels [16–18]. The conclusions regarding the diagnostic performance of next-generation sequencing (NGS) technology in comparison to commercially available genetic test panels remain controversial. Currently, there is a lack of literature that directly compares the two. In this study, we conducted a systematic review and meta-analysis to assess the diagnostic performance of molecular testing for indeterminate thyroid nodules. To ensure the accuracy of the results, we only included studies with pathologically confirmed diagnoses. Our investigation aims to identify study limitations and biases, while also exploring sources of variability in diagnostic accuracy across the included studies. Ultimately, this analysis seeks to provide a clearer understanding of the utility and reliability of molecular testing in clinical practice for managing thyroid nodules of uncertain malignant potential.

## Materials and methods

This article follows the PRISMA guidelines for systematic reviews and meta-analyses [19]. The datasets utilized in this meta-analysis were sourced exclusively from publicly accessible, peer-reviewed publications. All original studies included in this analysis had obtained prior approval from their respective Institutional Review Boards, ensuring compliance with ethical standards. As the data were fully anonymized and derived from previously published research, the requirement for additional informed consent was waived in accordance with established ethical guidelines for secondary data analysis.

This work was conducted by a multidisciplinary team of researchers with expertise and extensive training in endocrine surgery (XL, KVH, SVS, WH, DY), endocrinology (KVH, SVS, WH, DY), pathology (VM), and epidemiology (NB). The authors’ combined expertise ensures a comprehensive approach to data interpretation and enhances the reliability of the study findings.

### Data sources and searches

We conducted a systematic literature search in PubMed, Embase, Web of Science, and Cochrane Library databases. Detailed search strategies are provided in the ESM_1.doc.

### Inclusion and exclusion criteria

The inclusion criteria were developed using the PICOS framework (participants, interventions, comparators, outcomes, and study design). To qualify, studies had to meet the following criteria: (a) FNA cytology results must be classified using the Bethesda Thyroid Cytopathology Reporting System. (b) Studies must report molecular test results based on FNA samples in human patients, with surgical resection histopathology serving as the gold standard for nodule evaluation. (c) Primary studies should provide sufficient patient data, including details on true positives (TP), false positives (FP), true negatives (TN), false negatives (FN), postoperative pathological results, preoperative Bethesda classification, and the type and name of the genetic test used. (d) We included studies published from January 1st, 2018, till October 14th, 2024, to account for the 2018 update to international guidelines that incorporated genetic testing for indeterminate thyroid nodules. Case reports and case series with fewer than 10 cases were excluded. The term ‘clinical validity’ was defined as the capacity of a molecular test to accurately differentiate between patients with malignant and non-malignant thyroid nodules. The following studies were excluded: (a) Duplicates, reviews, comments, editorials, conference abstracts, and unpublished articles. (b) Studies lacking required data, such as those with missing surgical histopathology results. (c) Articles not published in English.

Two reviewers (XL, KVDH) independently screened the titles and abstracts of all articles for eligibility. Any disagreements were resolved by a third reviewer (NB).

### Data extraction and quality assessment

In addition to the necessary criteria mentioned above, optional information includes the article title, author names, publication year, number of nodules studied, classification of non-invasive follicular thyroid neoplasm with papillary-like nuclear features (NIFTP), country of the study, and study type (prospective or retrospective).

Risk of bias for each study was assessed independently by two reviewers (XL, KVDH) using explicit criteria from the Quality Assessment of Diagnostic Accuracy Studies-2 (QUADAS-2) tool [20]. Risk was categorized as low, moderate, or high, and any conflicts in assessment were resolved through discussion.

### Evaluation indicators

In this diagnostic meta-analysis, key statistical parameters were used to assess diagnostic accuracy: Negative Likelihood Ratio (NLR), Positive Likelihood Ratio (PLR), Diagnostic Odds Ratio (DOR), and Area Under the Curve (AUC) of the Receiver Operating Characteristic (ROC) curve. NLR and PLR are key indicators of a test’s diagnostic performance for malignant thyroid nodules. A lower NLR suggests the test can reliably exclude malignancy, while a higher PLR indicates its ability to accurately identify malignant nodules. DOR quantifies the overall discriminatory ability of a test, ranging from 0 to infinity, with a value of 1 signifying no diagnostic power and values above 10 indicating strong diagnostic accuracy. Additionally, the AUC ranges from 0 to 1.0, with values closer to 1.0 reflecting higher diagnostic accuracy and greater discriminatory capability. Sensitivity and specificity further highlight the test’s ability to differentiate between malignant and benign nodules.

### Data synthesis and statistical analysis

We used STATA^®^ (StataCorp, V.16·1/MP) to compute sensitivity, specificity, PLR, NLR, and DOR based on TP, FP, TN, and FN counts for each molecular panel. For combining data, we employed a bivariate random-effects model to estimate Sensitivity and specificity along with their corresponding 95% confidence intervals (CIs). To evaluate diagnostic performance, we constructed Summary Receiver Operating Characteristic (SROC) curves for each panel. AUC was utilized as a measure of diagnostic accuracy [21]. To assess heterogeneity among studies, we calculated I² statistics and employed Bivariate boxplot to identify potential outliers. We also assessed evidence of publication bias using Deek’s funnel plot [22]. Deek’s test bias test was performed, and the reliability was expressed as a 95% confidence interval (95% CI) [23]. 

## Results

### Study selection and inclusion

**Fig. 1:**
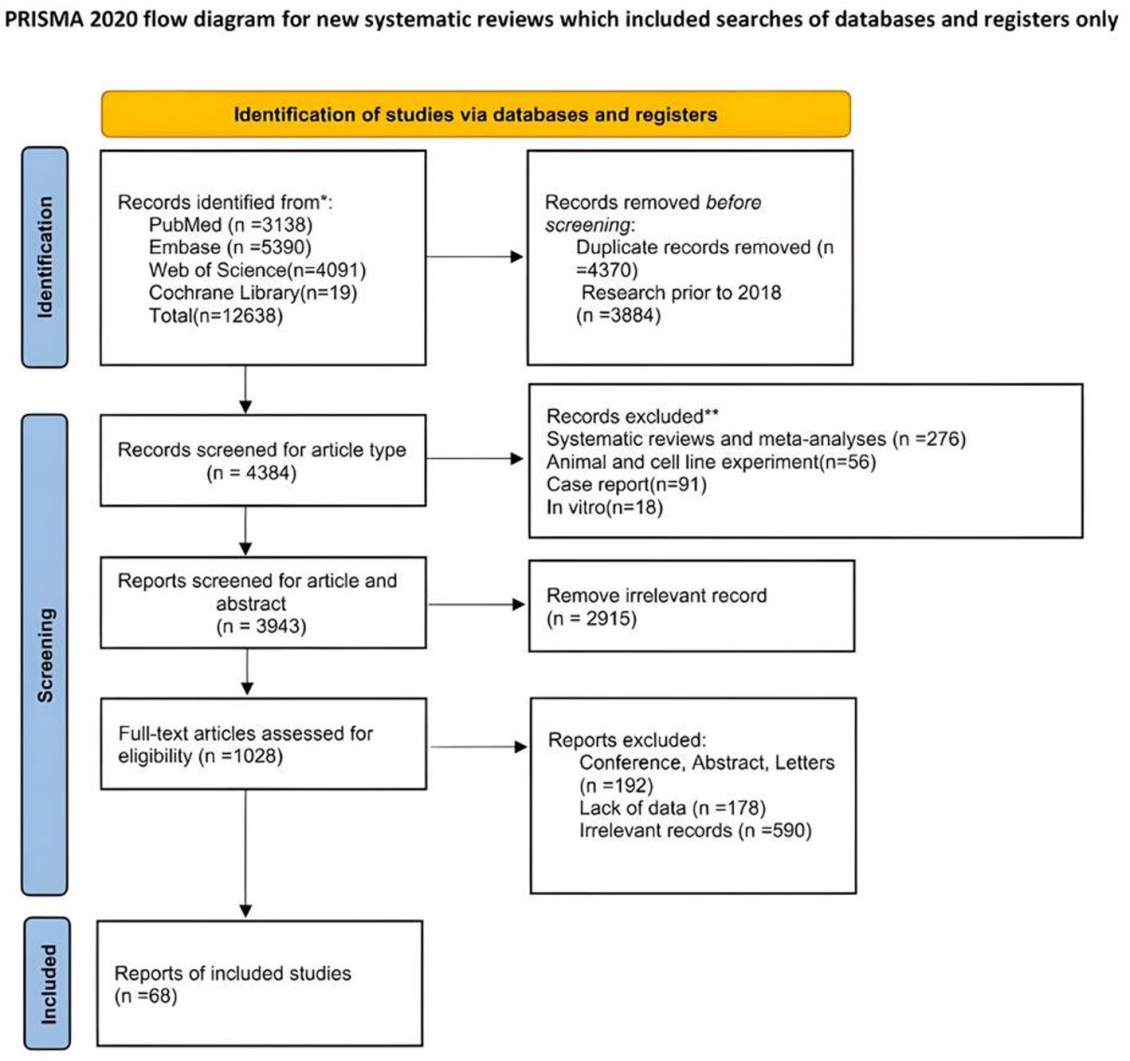
Preferred Reporting Items for Systematic Reviews and Meta-Analyses (PRISMA) flowchart of the included studies

In total, 68 papers were included (Fig. 1). Among these, 14 studies specifically assessed the clinical effectiveness of Afirma Gene Expression Classifier (GEC) [6, 24–36]. Thirteen studies evaluated Afirma GSC [24–26, 29, 30, 32–34, 36–40]. Seven studies evaluated both Afirma GEC and GSC [24, 25, 29, 30, 32, 33, 36]. Fifteen studies evaluated ThyroSeq v2 or v3 [6, 35, 41–53]. Three studies evaluated both ThyroSeq v2 and ThyroSeq v3 [42, 45, 51]. two studies evaluated Rosetta GX Reveal [31, 34], and 20 studies evaluated various types of Polymerase Chain Reaction (PCR) [54–73]. Five articles evaluated MPTX v1 [34, 74–77]. 

Of the 68 studies included, (Dataset 1), 18 used prospectively collected data [6, 28, 35, 38, 46, 55–57, 59, 60, 65, 66, 70, 71, 78–81]. NIFTP was reported as malignant in postoperative histopathologic findings in 31 studies [24–26, 28, 30, 38–41, 43–47, 52–55, 57, 59, 63, 64, 69, 74, 76, 77, 81–85]. Nine studies did not report NIFTP as malignant [6, 27, 29, 34, 37, 42, 50, 51, 86]. 

### Quality assessment

**Fig. 2:**
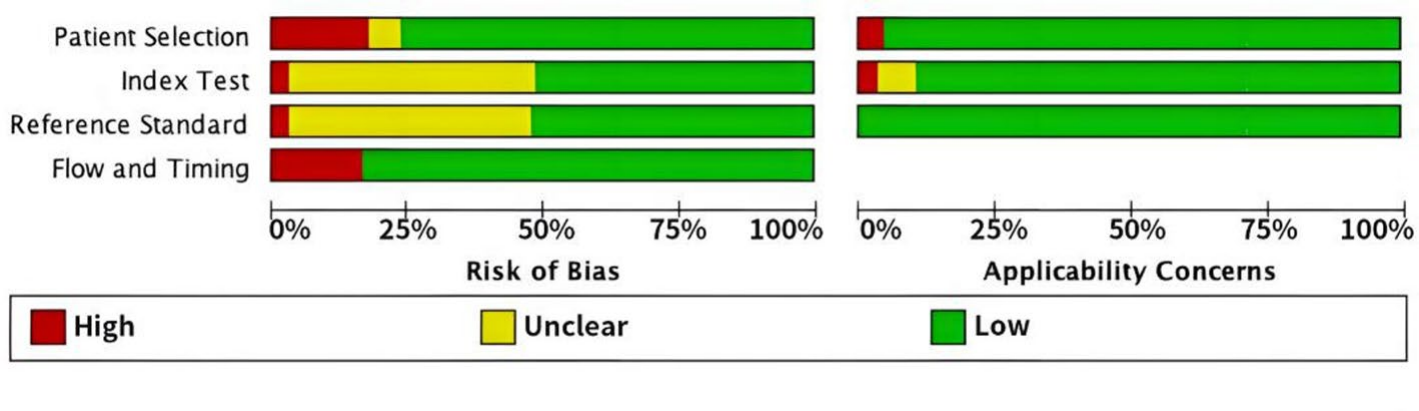
Summary of the risk of bias and applicability concerns assessed using the QUADAS-2 tool. Each bar represents the proportion of studies classified as having a low (green), unclear (yellow), or high (red) risk of bias in each domain. The assessment includes patient selection, index test, reference standard, and flow and timing

The assessment included a methodological quality graph and summary (Fig. 2), highlighting the findings on risk of bias and applicability concerns. Over 50% of the articles were classified as having a low risk of bias, indicating that the overall quality of the included studies is high.

### Diagnostic performance of Afirma GEC and GSC

**Fig. 3:**
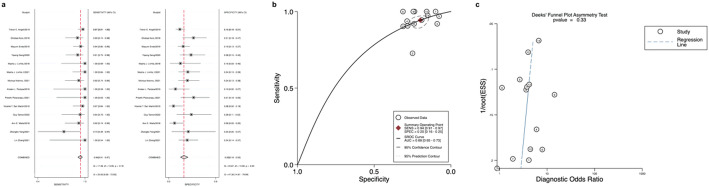
**a**: Forest plot of sensitivity and specificity for Afirma Gene Expression Classifier (GEC); **b**: Afirma GEC Overall Sensitivity and Specificity Summary Receiver Operating Characteristic (SROC Curve); **c**: Afirma GEC Deek’s funnel plot asymmetry test for publication bias

The forest plot (Fig. 3a) shows a Sensitivity of 0.94 and a specificity of 0.20. Inter-study heterogeneity was low. The pooled results for the Afirma GEC showed a PLR of 1.2 (95% CI: 1.1–1.2), a NLR of 0.28 (95% CI: 0.18–0.45), a summary of the SROC curve was 0.69 (95% CI: 0.65–0.73) (Fig. 3b). The overall DOR for Afirma GEC was 4 (95% CI: 2–7) (Table 1).

**Table 1:** A review of the diagnostic performance of molecular tests for the diagnosis of indeterminate thyroid nodules (cytological results of Bethesda III, IV, and V)

Panel	Afirma GEC	Afirma GSC	Thyroseq v2	Thyroseq v3	NGS^a^	MPTX v1
No of studies	14	8	9	8	9	4
SEN^b^ [95%CI]	0.94[0.90–0.97]	0.94[0.88–0.97]	0.91[0.85–0.94]	0.91[0.87-o.94]	0.75[0.69–0.80]	0.94[0.84–0.98]
SPEC^c^ [95%CI]	0.21[0.16–0.26]	0.36[0.27–0.46]	0.52[0.46–0.57]	0.42[0.36–0.49]	0.72[0.64–0.80]	0.55[0.45–0.64]
PLR^d^ [95%CI]	1.2[1.1–1.2]	1.5[1.3–1.8]	1.9[1.6 − 0.2.1]	1.6[1.4–1.8]	2.7[2.0-3.6]	2.1[1.6–2.6]
NLR^e^ [95%CI]	0.28[0.18–0.45]	0.18[0.09–0.35]	0.18[0.11–0.31]	0.21[0.14–0.30]	0.35[0.28–0.43]	0.12[0.04–0.33]
DOR^f^ [95%CI]	4[2–7]	8[4–18]	10[5–20]	8[5–12]	8[5–12]	18[5–60]
AUC^g^	0.69[0.65–0.73]	0.89[0.86–0.92]	0.69[0.65–0.73]	0.76[0.72–0.80]	0.78[0.75–0.82]	0.75[0.71–0.78]

^a^Next-Generation Sequencing

^b^Sensitivity

^c^Specificity

^d^Positive predictive value

^e^Negative likelihood ratio

^f^Diagnostic odds ratio

^g^area under the curve

**Fig. 4:**
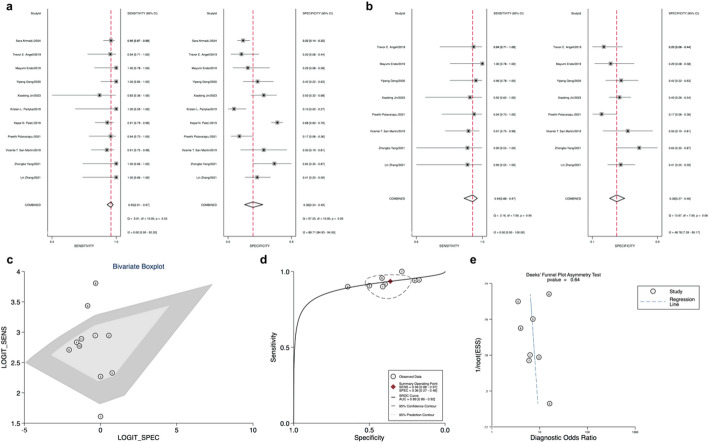
**a**: Forest plot of sensitivity and specificity for Gene Sequencing Classifier (GSC); **b**: Forest plot of overall sensitivity and specificity of GSC, excluding excluded studies; **c**: Bivariate boxplot for GSC; **d**: GSC Overall Sensitivity and Specificity Summary Receiver Operating Characteristic(SROC Curve) (excluding excluded articles); **e**: GSC Deek’s funnel plot asymmetry test for publication bias

Eleven studies evaluated the Afirma GSC panel. The forest plot (Fig. 4a) demonstrated high heterogeneity in Sensitivity (I²=0.00, 95% CI: 0 to 97.74) and Specificity (I²=89.71, 95% CI: 84.93–94.5), necessitating the application of a random-effects model for meta-analysis. Outliers identified by bivariate box plot (Fig. 4c) were excluded from the comprehensive analysis of Afirma GSC [34, 38, 40]. The Sensitivity was 0.94 (95% CI: 0.88–0.97), Specificity was 0.36 (95% CI: 0.27–0.46), and AUC was 0.89 (95% CI: 0.86–0.92), as depicted in Fig. 4b and d. Upon excluding three studies, the meta-analysis of Afirma GSC yielded a PLR of 1.5 (95% CI: 1.3–1.8), NLR of 0.18 (95% CI: 0.09–0.35), and DOR of 8 (95% CI: 4–18) (Table 1).

Deek’s funnel plots assessed publication bias across all 14 studies included in our meta-analysis of Afirma GEC, showing no significant bias (*p* = 0.33 for Deek’s funnel plot asymmetry test) (Fig. 3c). Similarly, there was also no publication bias in the GSC among the 8 studies included (Deek’s funnel plot asymmetry test *p* = 0.64) (Fig. 4e).

### Diagnostic performance of Thyroseq v2 and v3

**Fig. 5:**
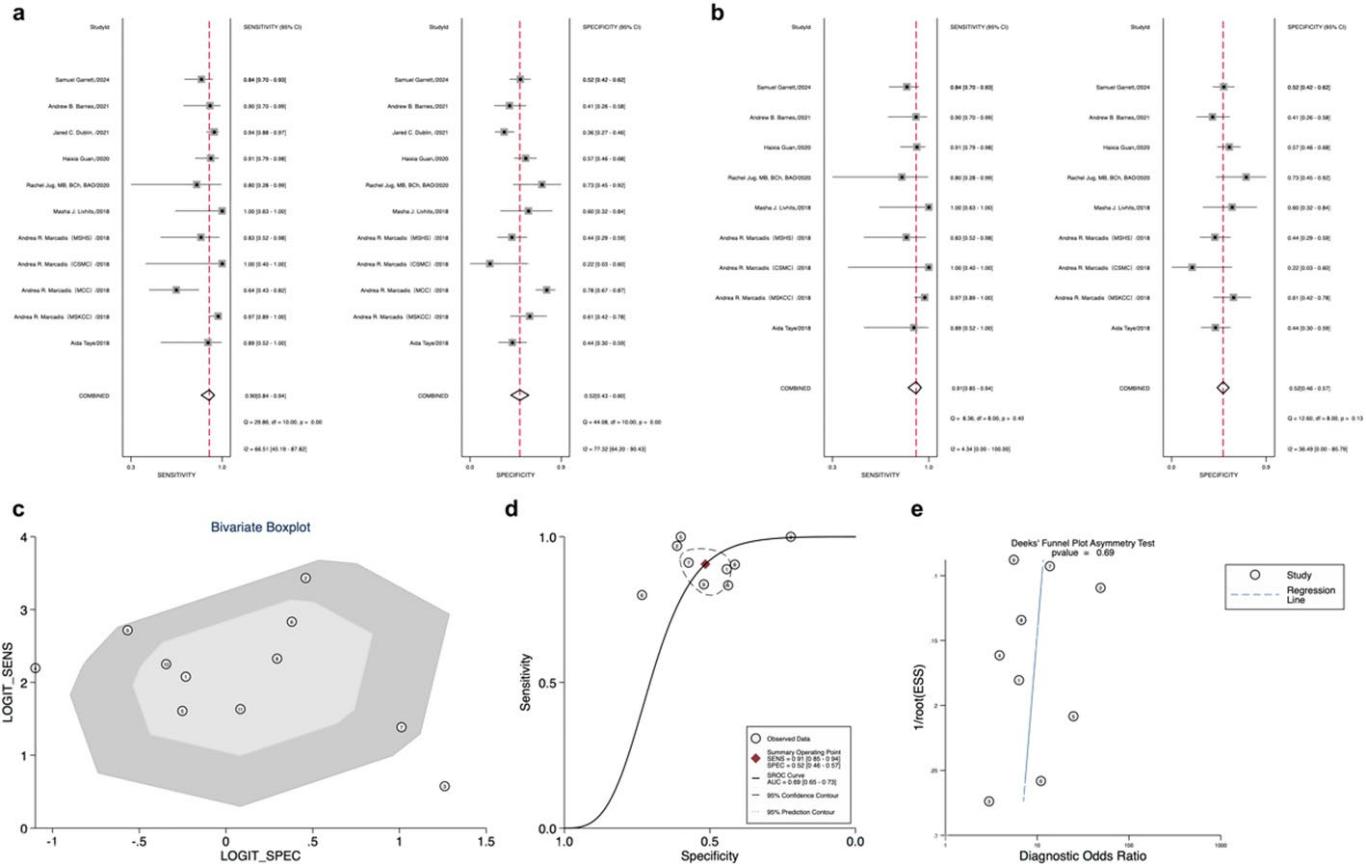
**a** Forest plot of sensitivity and specificity for Thyroseq version 2; **b**: Forest plot of overall sensitivity and specificity of Thyroseq v2, excluding excluded studies; **c**: Bivariate boxplot for Thyroseq v2; **d**: Thyroseq v2 Overall Sensitivity and Specificity Summary Receiver Operating Characteristic(SROC Curve) (excluding excluded articles); **e**: Thyroseq v2 Deek’s funnel plot asymmetry test for publication bias

A total of eleven articles on ThyroSeq v2 were included in our study, with one of them being a multicenter study, each contributing as an independent sample. The forest plot (Fig. 5a) revealed significant heterogeneity among studies for sensitivity (I² = 66.51%, 95% CI: 49.19 to 81.82) and SP (I² = 77.32%, 95% CI: 64.20 to 90.43). we conducted a sensitivity analysis series and generated bivariate box plot graphs (Fig. 5c). We excluded 2 articles with high heterogeneity [44, 49]. In our meta-analysis, the combined analysis for ThyroSeq v2 yielded a sensitivity of 0.91 (95% CI: 0.85–0.94), specificity of 0.52 (95% CI: 0.46–0.57), and AUC of 0.69 (95% CI: 0.65–0.73) (Fig. 5b and d). PLR was 1.9 (95% CI:1.6–2.1), the NLR was 0.18 (95% CI: 0.11–0.31), and DOR was 10 (95% CI: 5–20) (Table 1).

**Fig. 6:**
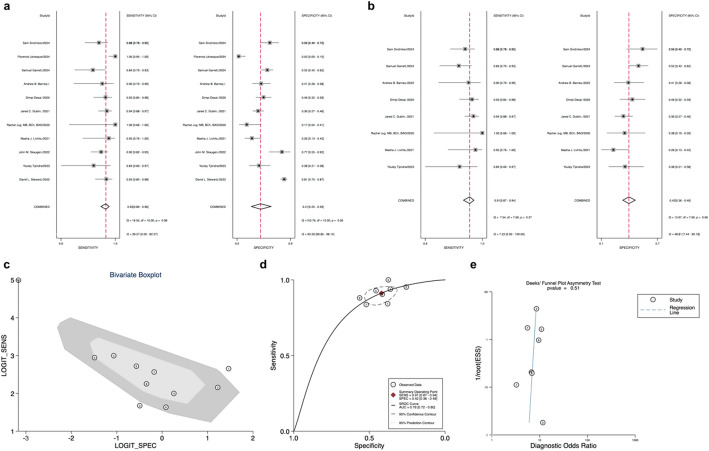
**a**: Forest plot of sensitivity and specificity for Thyroseq version 3; **b**: Forest plot of overall sensitivity and specificity of Thyroseq v3, excluding excluded studies; **c**: Bivariate boxplot for Thyroseq v3; **d**: Thyroseq v3 Overall Sensitivity and Specificity Summary Receiver Operating Characteristic(SROC Curve) (excluding excluded articles); **e**: Thyroseq v3 Deek’s funnel plot asymmetry test for publication

Eleven articles evaluating ThyroSeq v3 were included in our study, as depicted in Fig. 6a. We observed high heterogeneity for sensitivity (I² = 39.57, 95% CI: 0 to 82.37) and specificity (I² = 93.50%, 95% CI: 90.85 to 96.14). For ThyroSeq v3, after performing a sensitivity analysis series and excluding three studies identified as outliers by bivariate box plot (Fig. 6c), we conducted a pooled analysis [41, 46, 52]. Sensitivity was 0.91 (95% CI: 0.87–0.94), specificity was 0.42 (95% CI: 0.36–0.49), and AUC was 0.76 (95% CI: 0.72–0.8) (Fig. 6b and d). PLR was 1.6 (95% CI: 1.4–1.8), NLR was 0.21 (95% CI: 0.14–0.30), and DOR was 8 (95% CI: 5–12) (Table 1).

Deek’s funnel plots, assessing publication bias, showed no evidence of bias for ThyroSeq v2 (*p* = 0.69 for Deek’s funnel plot asymmetry test) (Fig. 5e) and ThyroSeq v3 (*p* = 0.51 for Deek’s funnel plot asymmetry test) (Fig. 6e) based on all studies included in our meta-analysis.

### Diagnostic performance of NGS and MPTX v1

**Fig. 7:**
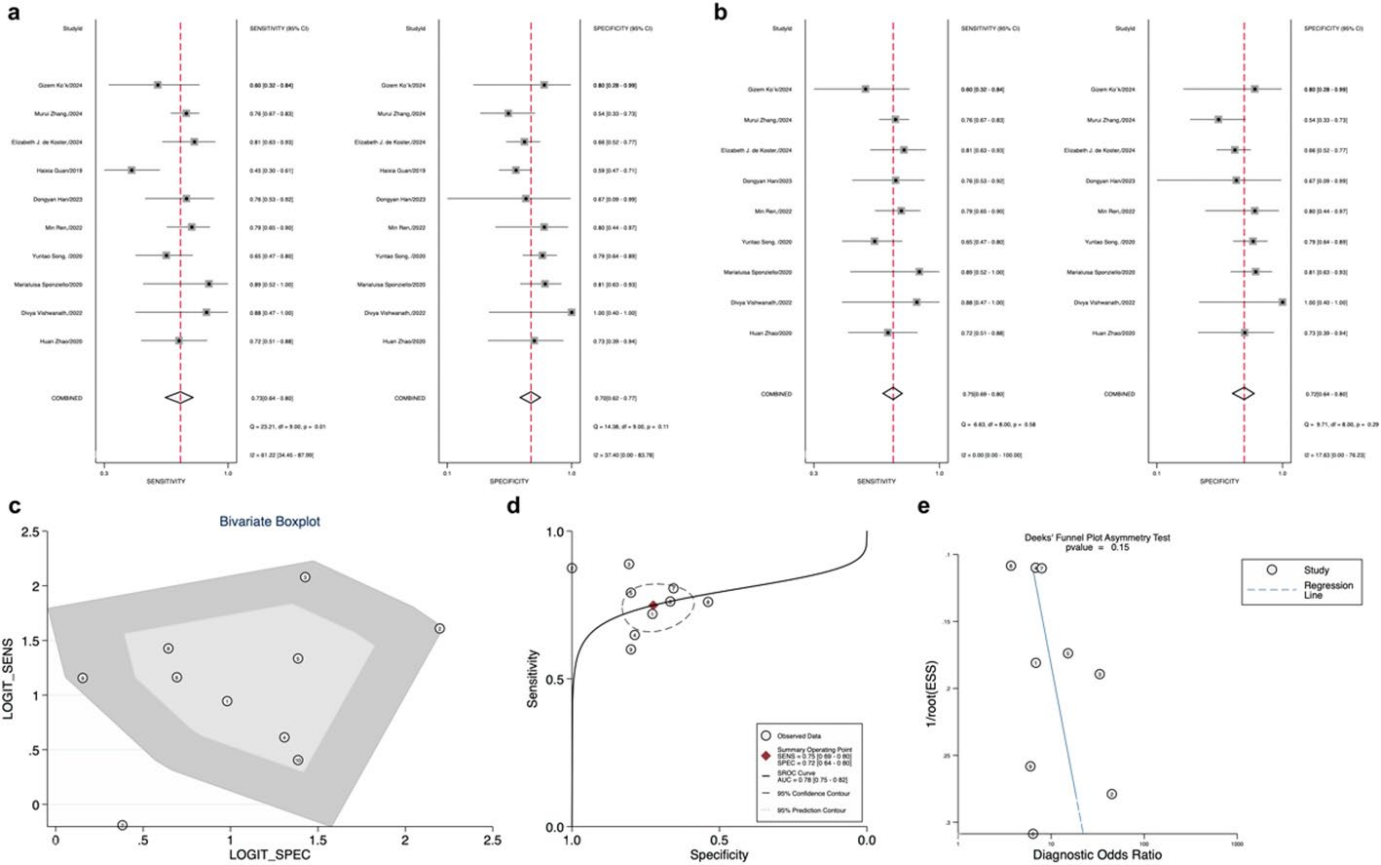
**a**: Forest plot of sensitivity and specificity for Next-Generation Sequencing (NGS); **b**: Forest plot of overall sensitivity and specificity of NGS, excluding excluded studies; **c**: Bivariate boxplot for NGS; **d**: NGS Overall Sensitivity and Specificity Summary Receiver Operating Characteristic ༈SROC Curve༉ (excluding excluded articles); **e**: NGS Deek’s funnel plot asymmetry test for publication

**Fig. 8:**
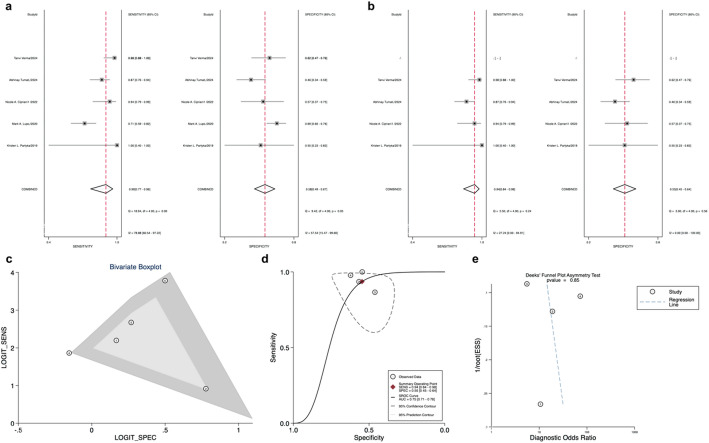
**a**: Forest plot of sensitivity and specificity for Multigene Point-of-care Test v1(MPTX v1); **b**: Forest plot of overall sensitivity and specificity of MPTX v1 excluding excluded studies; **c**: Bivariate boxplot for NGS; **d**: MPTX v1 Overall Sensitivity and Specificity Summary Receiver Operating Characteristic (SROC Curve) (excluding excluded articles); **e**: MPTX v1 Deek’s funnel plot asymmetry test for publication

Ten articles on NGS were included in this study. The forest plot showed significant heterogeneity (Fig. 7a). After excluding 1 independent study [82], the results of the combined analysis were sensitivity 0.75 (95% CI: 0.69–0.80), specificity 0.72 (95% CI: 0.64–0.80), and AUC 0.78 (95% CI: 0.75–0.82) (Fig. 7b and d), and the bivariate boxplot is shown in Fig. 7c. Five articles on MPTX v1 were included in this study. The forest plot showed significant heterogeneity (Fig. 8a). After excluding 1 independent study [74], the results of the combined analysis were low heterogeneity, and the bivariate boxplot is shown in Fig. 8c. The results of the combined analysis of sensitivity, specificity, and AUC are in Fig. 8b and d.

Deek’s plots of all 9 studies included in our meta-analysis showed we had no publication bias for NGS (Fig. 7e). There was no publication bias for MPTX v1 (Fig. 8e).

The PCR group could not be analyzed by meta-analysis due to large differences in methodological heterogeneity across studies (Dataset 2). This study was difficult to undertake for the other molecular tests too, since very few studies were available.

## Discussion

Molecular testing has emerged as a critical tool in the diagnosis of indeterminate thyroid nodules, particularly in cases where cytological results are inconclusive or ambiguous, offering enhanced diagnostic accuracy and guiding clinical decision-making [1, 8]. These tests play a crucial role in guiding treatment decisions; however, currently available molecular panels still have limitations in accurately distinguishing benign from malignant thyroid nodules. Several studies have evaluated their diagnostic performance, reporting varying levels of sensitivity and specificity [8, 17]. In this meta-analysis, we provide a comprehensive assessment of widely used molecular assays in clinical practice, including Afirma GEC, GSC, Thyroseq v2, Thyroseq v3, and NGS.

Our findings indicate that the Afirma assays exhibit the highest sensitivity but relatively lower specificity, making them less reliable for ruling out benign nodules. In contrast, Thyroseq v2 demonstrated a more balanced performance, with significantly improved specificity while maintaining high sensitivity. Thyroseq v3 showed a slight improvement in sensitivity; however, this came at the cost of reduced specificity. Interestingly, these results differ from earlier studies reporting higher sensitivity and specificity for these assays. This discrepancy may be due to differences in sample selection, patient characteristics, and analytical methods across the included studies [9, 17, 87–90]. 

Our data also imply that in a surgically confirmed cohort other molecular tests such as the 7-gene mutation panel (BRAF, RAS, RET/PTC, and PAX8/PPARγ) may have higher specificity compared to Afirma or Thyroseq, however at the cost of reduced sensitivity. This finding underscores the potential clinical utility of mutation-based panels when reducing false-positive diagnoses is considered critical, for example, in preventing unnecessary thyroidectomy in patients with indeterminate nodules. Highlighting this trade-off between sensitivity and specificity is important to guide clinicians’ choice on the use of molecular tests, according to patient risk profile. Regarding the inclusion of Bethesda V nodules, although these lesions are classified as “suspicious for malignancy,” a substantial proportion are ultimately benign, with reported malignancy rates ranging from 45% to 60%. We included Bethesda V nodules to provide a comprehensive evaluation of molecular testing across the full spectrum of cytologically indeterminate thyroid nodules, which also offers clinically valuable information to guide surgical decision-making and may help avoid unnecessary thyroidectomy in nodules that are ultimately benign.

This study highlights the critical role of postoperative pathological examination as the gold standard for thyroid nodule diagnosis. By ensuring the highest level of diagnostic accuracy, this approach minimizes the biases associated with imaging and cytology. Furthermore, restricting the analysis to surgically confirmed cases help reduce misclassification due to indeterminate cytological results and mitigates heterogeneity arising from varying diagnostic criteria.

Significant variability was observed across studies, particularly in specificity for GSC, Thyroseq v2, Thyroseq v3, and NGS. This heterogeneity may stem from differences in patient demographics, clinical settings, and testing methodologies. However, the lack of detailed patient data in many included studies limited our ability to explore these factors comprehensively. Although sensitivity analyses accounted for some potential sources of variability, their influence may still impact the robustness of our conclusions.

Among the evaluated assays, MPTX v1, a multiplatform mutation panel combined with a microRNA risk classifier, demonstrated the lowest NLR, making it particularly effective in ruling out malignancy. Its high sensitivity and specificity suggest that it may reduce unnecessary surgeries and improve overall diagnostic accuracy. However, further validation is required to confirm its clinical utility across broader populations.

This meta-analysis has several limitations. First, our inclusion criteria required surgical histopathologic confirmation, which may have inflated the positive predictive value (PPV) and sensitivity, as the study cohorts were enriched with confirmed malignancies. Second, insufficient descriptions of surgical samples in some studies complicated the interpretation of results, highlighting the need for more standardized reporting in future research.

Moving forward, future studies should focus on linking molecular testing outcomes with long-term clinical endpoints, such as recurrence rates and overall survival. Longitudinal studies with diverse populations and Bethesda category stratifications will be essential to refining clinical guidelines and optimizing the use of molecular assays. Advances in molecular testing technologies hold promise for enhancing diagnostic precision while minimizing unnecessary surgical interventions and healthcare costs.

Some molecular assays, such as MPTX v2 and RosettaGX Reveal, lacked sufficient data for inclusion in this meta-analysis. Further studies are needed to validate their diagnostic performance and determine their potential role in clinical practice.

Active surveillance for indeterminate thyroid nodules is a relatively low-cost approach. Patients with clinically and ultrasonographically benign features typically require only periodic blood tests and thyroid ultrasounds every 6 to 12 months, with an estimated cost of $100 to $200 [91–93]. While molecular tests such as Afirma GEC ($4,875) and ThyroSeq ($3,200) entail higher upfront costs, they may help mitigate the overall financial impact by reducing the risk of delayed diagnosis and associated complications [92, 93]. This suggests that genetic testing can effectively reduce patient costs by preventing missed diagnoses and unnecessary treatments.

While no single molecular test can fully distinguish between benign and malignant thyroid nodules, each method has its strengths. Our results demonstrate that Thyroseq v2 and NGS show high diagnostic accuracy, with Thyroseq v2 offering better sensitivity than v3. MPTX v1 is even more effective in identifying malignant thyroid nodules. Nevertheless, the overall diagnostic performance of these molecular assays remains suboptimal, emphasizing the need for ongoing refinement and rigorous validation.

## Supplementary Information

Below is the link to the electronic supplementary material.

Supplementary Material 1

Supplementary Material 2

Supplementary Material 3

## Data Availability

All data used in this study were extracted from previously published articles and are available in the supplementary files provided with this manuscript. Further information is available from the corresponding author upon reasonable request.
