# Supplementary material for: Comparing the diagnostic accuracy of pre-operative genetic testing for thyroid cancer on fine needle aspiration cytology specimens: a systematic review and meta-analysis of diagnostic accuracy
Source: Discov Oncol. 2025 Oct 2;16:1797. doi: 10.1007/s12672-025-03676-9 (PMC12491129; doi:10.1007/s12672-025-03676-9)
Supplement: Supplementary file 2 — Supplementary Material 2 [file 12672_2025_3676_MOESM2_ESM.docx]

**Supplementary material**

**Comparing the Diagnostic Accuracy of pre-operative Genetic Testing for Thyroid Cancer on Fine Needle Aspiration Cytology Specimens: A Systematic Review and Diagnostic Accuracy Meta-analysis**

Xin LIAO ^1^ , Klaas VAN DEN HEEDE ^2^ , Bruno LAPAUW ^3^ , Wouter HUVENNE ^4^ , Dirk YSEBAERT ^5^ , Sam VAN SLYCKE ^2^ , Nele BRUSSELAERS ^1,7,8^

Correspondence: [Xin.Liao@student.uantwerpen.be](mailto:Xin.Liao@student.uantwerpen.be)

**Supplement 1: Systematic literature search: search strings, data extraction and reasons and references for removal by full text**

We followed the PRISMA-Search checklist and utilized a flowchart (Figure 1) to present our study selection process. Original studies were defined as observational or experimental studies (trials) that included quantitative data. Specifically, we included observational studies (e.g., case-control and cohort studies) and experimental studies that provided primary genetic testing data.

The study population consisted of patients with thyroid nodules who underwent ultrasound evaluation and fine-needle aspiration (FNA), from whom tissue samples were collected for genetic analysis. Key genetic alterations, such as mutations in *BRAF*, *TERT*, and *RAS*, as well as gene expression characteristics, were assessed. The genetic testing results were compared with pathological diagnoses (the "gold standard"), and diagnostic performance metrics—including sensitivity, specificity, and receiver operating characteristic (ROC) curves—were analyzed.

The study excluded animal experiments, systematic reviews, letters, and opinion pieces lacking quantitative assessments. Conference abstracts were only included when corresponding full-text articles were unavailable. Only English-language studies were considered. Articles reporting molecular testing results without cytological classification were deemed insufficient and excluded.

Relevant studies were screened using EndNote 21 by two independent authors (XL, KVDH). Extracted data included authors, publication year, study design, type of genetic testing, molecular markers analyzed, country of origin, cytological results, and whether noninvasive follicular thyroid neoplasm with papillary-like nuclear features (NIFTP) was classified as malignant.

| **Database** | **Search string** | **Date of last search** | **Number of articles** |
| --- | --- | --- | --- |
| PubMed | ("Genetic Testing"[MeSH Terms] OR"gene*"[TIAB] OR "afirma gene expression classifier"[TIAB] OR"GEC"[TIAB] OR "ThyroSeq"[TIAB] OR "ThyGenX"[TIAB] OR"ThyraMIR"[TIAB] OR (("rosetta"[All Fields] OR "rosetta s"[All Fields]) AND "GX"[TIAB]) OR "afirma gene sequencing classifier"[TIAB] OR "xpression atlas"[TIAB] OR (("multiplatform"[All Fields] AND ("mutate"[All Fields] OR "mutated"[All Fields] OR "mutates"[All Fields] OR "mutating"[All Fields] OR "mutation"[MeSH Terms] OR "mutation"[All Fields] OR "mutations"[All Fields] OR "mutation s"[All Fields] OR "mutational"[All Fields] OR "mutator"[All Fields] OR "mutators"[All Fields]) AND ("panel"[All Fields] OR "panel s"[All Fields] OR "panels"[All Fields]) AND ("microrna s"[All Fields] OR "micrornas"[MeSH Terms] OR "micrornas"[All Fields] OR "microrna"[All Fields]) AND ("risk"[MeSH Terms] OR "risk"[All Fields]) AND "classifier test"[TIAB]) OR (("receptors, thyrotropin"[MeSH Terms] OR ("receptors"[All Fields] AND "thyrotropin"[All Fields]) OR "thyrotropin receptors"[All Fields] OR ("thyrotropin"[All Fields] AND "receptor"[All Fields]) OR "thyrotropin receptor"[All Fields]) AND "mrna test"[TIAB]) OR "TSHR"[TIAB] OR "mRNA"[TIAB] OR "RNA"[TIAB] OR "liquid biopsy"[TIAB] OR "liquid biopsies"[TIAB] OR "DNA"[TIAB] OR "cell-free"[TIAB] OR "cell-free"[TIAB] OR "nucleic acids"[TIAB] OR "mutation*"[TIAB] OR "cfDNA"[TIAB] OR "cirDNA"[TIAB] OR "deoxyribonucleic acid"[TIAB] OR "ribonucleic acid"[TIAB] OR "cfRNA"[TIAB] OR "cirRNA"[TIAB] OR "sequenc*"[TIAB] OR "panel*"[TIAB] OR "Expression"[TIAB]) AND ("thyroid nodule"[MeSH Terms] OR "nodule*"[TIAB] OR "Bethesda"[TIAB] OR "atypia of undetermined significance"[TIAB] OR "AUS"[TIAB] OR "follicular lesion of undetermined significance"[TIAB] OR "FLUS"[TIAB] OR "Follicular"[TIAB] OR "malignan*"[TIAB] OR "cancer"[TIAB] OR "tumor*"[TIAB] OR"neoplasm*"[TIAB] OR "Undetermined"[TIAB] OR "lesion*"[TIAB] OR "carcino*"[TIAB]) AND "Thyroid"[TIAB] AND ("sensitiv*"[TIAB] OR "sensitivity and specificity"[MeSH Terms] OR ("predictive"[TIAB] AND "value*"[TIAB]) OR "predictive value of tests"[MeSH Terms] OR "accuracy*"[TIAB]) AND 2009/01/01:2024/12/31[Date - Publication] | Oct 14, 2024 | 3138 |
| Web of Science | Search: ((((((((((((((((((((((((TS=(genetic screening)) OR TS=(predictive genetic testing)) OR TS=(genetic predisposition testing)) OR TS=(afirma gene expression classifier)) OR TS=(gec)) OR TS=(thyroseq)) OR TS=(thygenx)) OR TS=(thyramir)) OR TS=(rosetta)) OR TS=(afirma gene sequencing classifier)) OR TS=(xpression atlas)) OR TS=(mutates)) OR TS=(microrna’,‘classifier)) OR TS=(mrna test)) OR TS=(tshr)) OR TS=(mrna)) OR TS=(rna)) OR TS=(liquid biopsy)) OR TS=(dna)) OR TS=(cfdna)) OR TS=(cirdna)) OR TS=(cfrna)) OR TS=(cirrna)) OR TS=(sequenc*)) OR TS=(panel)) AND (((TS=(sensitivity and specificity)) OR TS=(sensitiv*)) OR TS=(predictive)) OR TS=(value)) OR TS=(predictive value of tests)) OR TS=(accuracy*)) AND (((TS=(thyroid nodule*)) OR TS=(atypia of undetermined significance)) OR TS=(follicular lesion of undetermined significance)) OR TS=(follicular)) OR TS=(undetermined)) OR TS=(thyroid neoplasms)) OR TS=(bethesda) | Oct 14, 2024 | 4091 |
| Embase | 'sensitivity and specificity'/exp OR 'sensitivity and specificity' AND 'sensitiv*':ti,ab AND 'predictive':ti,ab AND 'value*':ti,ab AND 'predictive value of tests':ti,ab AND 'accuracy*':ti,ab AND 'undetermined':ti,ab AND 'follicular':ti,ab AND 'malignant':ti,ab AND 'benign':ti,ab AND 'neoplasm':ti,ab AND 'cancer':ti,ab AND 'nodule':ti,ab AND 'tumor':ti,ab AND 'cytology':ti,ab AND 'diagnosis':ti,ab AND 'pathology':ti,ab AND 'genetic':ti,ab AND 'risk':ti,ab AND 'screening':ti,ab AND 'diagnostic':ti,ab AND 'test':ti,ab AND 'biopsy':ti,ab AND 'analysis':ti,ab AND 'mutation':ti,ab AND 'study':ti,ab AND 'clinical':ti,ab AND 'evaluation':ti,ab AND 'results':ti,ab AND 'sensitivity':ti,ab AND 'specificity':ti,ab AND 'predictive':ti,ab AND 'accuracy':ti,ab AND 'thyroid nodule'/exp OR 'thyroid nodule' AND 'atypia of undetermined significance':ti,ab AND 'follicular lesion of undetermined significance':ti,ab AND 'thyroid neoplasms':ti,ab AND 'thyroid nodules':ti,ab AND 'bethesda':ti,ab |  | 5390 |
| Cochrane | (MeSH descriptor: [Thyroid Nodule] explode all trees OR ("thyroid nodules"):ti,ab,kw OR ("thyroid neoplasm"):ti,ab,kw OR ("Bethesda"):ti,ab,kw OR (atypia of undetermined significance):ti,ab,kw OR (follicular lesion of undetermined significance):ti,ab,kw) AND (MeSH descriptor: [Genetic Testing] explode all trees OR ("genetic screening"):ti,ab,kw OR ("predictive genetic testing"):ti,ab,kw OR ("genetic predisposition"):ti,ab,kw OR ("afirma gene expression classifier"):ti,ab,kw OR (throseq):ti,ab,kw OR (GEC):ti,ab,kw OR ("gsc"):ti,ab,kw OR ("sequence"):ti,ab,kw OR (panel):ti,ab,kw AND (rosetta):ti,ab,kw OR (thyramir):ti,ab,kw OR (rosetta):ti,ab,kw OR (xpression atlas):ti,ab,kw OR (sequenc*):ti,ab,kw OR (panel):ti,ab,kw) AND ("sensitivity and specificity" OR ("sensitive"):ti,ab,kw OR ("predictive accuracy"):ti,ab,kw OR (value):ti,ab,kw OR ("predictive value of tests"):ti,ab,kw AND ("predictive"):ti,ab,kw) | Oct 14, 2024 | 19 |
|  |  | Total | 12638 |
